# Prolonged survival in patients with local chronic infection after high-grade glioma treatment: Two case reports

**DOI:** 10.3389/fonc.2022.1073036

**Published:** 2022-12-16

**Authors:** Peter Solár, Zdenek Mackerle, Michal Hendrych, Petr Pospisil, Radek Lakomy, Hana Valekova, Marketa Hermanova, Radim Jancalek

**Affiliations:** ^1^ Department of Neurosurgery, St. Anne’s University Hospital Brno, Brno, Czechia; ^2^ Department of Neurosurgery, St. Anne’s University Hospital Brno, Faculty of Medicine, Masaryk University, Brno, Czechia; ^3^ First Department of Pathology, St. Anne’s University Hospital Brno, Brno, Czechia; ^4^ First Department of Pathology, St. Anne’s University Hospital Brno, Faculty of Medicine, Masaryk University, Brno, Czechia; ^5^ Department of Radiation Oncology, Masaryk Memorial Cancer Institute, Faculty of Medicine, Masaryk University, Brno, Czechia; ^6^ Department of Comprehensive Cancer Care, Masaryk Memorial Cancer Institute, Faculty of Medicine, Masaryk University, Brno, Czechia

**Keywords:** high-grade glioma, glioblastoma, anaplastic astrocytoma, wound infection, prolonged survival

## Abstract

High-grade gliomas are primary brain tumors with poor prognosis, despite surgical treatment followed by radiotherapy and concomitant chemotherapy. We present two cases of long-term survival in patients treated for high-grade glioma and concomitant prolonged bacterial wound infection. The first patient treated for glioblastoma IDH-wildtype had been without disease progression for 61 months from the first resected recurrence. Despite incomplete chemotherapy-induced myelosuppression in the second patient with anaplastic astrocytoma IDH-mutant, she died without disease relapse after 14 years from the diagnosis due to other comorbidities. We assume that the documented prolonged survival could be related to the bacterial infection.

## 1 Introduction

High-grade gliomas (HGG) are primary brain tumors with relatively high incidence and poor prognosis. Despite surgical treatment followed by radiotherapy and concomitant chemotherapy, the median overall survival (OS) time is approximately 14-16 months in patients with glioblastoma (GBM) IDH-wildtype, WHO grade IV and 3 to 5 years for anaplastic astrocytoma (AA) IDH-mutant, WHO grade III (1, 2). Considering the low effectiveness of the current standard of care, new possibilities in GBM treatment have been investigated. Immunotherapy seems to be one of the potential therapeutic approaches in treating HGG (3). Based on GBM anti-tumor response, several immunotherapies, including adoptive cell therapy, immune-virotherapy, dendritic-cell-based therapy, and peptide vaccination to stimulate the immune response, are being investigated (4). One of the potential activators of the immune response may also be a bacterial infection. It was found that bacterial-based tumor therapy inhibits tumor cell growth in different cancer types, like sarcomas or superficial bladder cancer (5, 6). Here we present two case reports of unexpectedly long survival in patients with chronic and prolonged bacterial infection at the site of craniotomy after standard therapy of HGG. We also discuss the possible impact of postoperative infection or other related factors on survival in patients with HGG.

## 2 Case report 1

**Figure 1:**
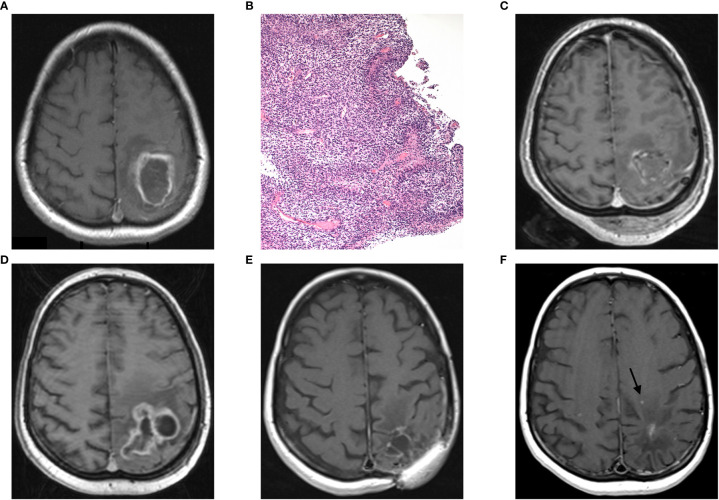
Case report 1: **(A)** preoperative axial MRI (postcontrast T1 weighted image) showing an intra-axial contrast-enhancing lesion in the left parietal lobe with perilesional edema extending to the central region, **(B)** histopathological finding of hypercellular pleomorphic glial neoplasia with multiple palisading necroses and glomeruloid microvascular proliferations (H&E, original magnification 100x), **(C)** postoperative MRI showing subtotal tumor resection, **(D)** GBM recurrence 5 months after primary tumor resection, **(E)** 2 years follow-up MRI, and **(F)** MRI with second tumor recurrence (arrow) 61 months after the first GBM recurrence.

A 45–year old female suffering from right hemiparesis and tactile hemi-hypesthesia was referred to our neurosurgery department with an intra-axial contrast-enhancing lesion in the left parietal lobe with perilesional edema extending to the central region (**Figure 1A**). The patient underwent 5-ALA fluorescence-guided subtotal tumor resection with electrophysiological monitoring and the histopathological diagnosis of GBM IDH-wildtype, WHO grade IV MGMT promotor methylated (**Figures 1B, C**). Her clinical status started to improve; however, the follow-up MRI in 3 months after concurrent chemoradiotherapy and adjuvant chemotherapy according to the original Stupp’s protocol revealed an early GBM progression (**Figure 1D**). Because of good quality of life, a second surgery using the same technique was performed and the result was classified as a gross total resection of GBM recurrence. Postoperatively, the patient underwent 7 cycles of palliative second-line chemotherapy with lomustine monotherapy (110 mg/m2) in a 6-week schedule followed by an MRI every 3 months.

A year after the second surgery, the patient was checked by a neurosurgeon due to a purulent secretion classified as chronic osteomyelitis caused by Staphylococcus aureus infection at the site of the surgical approach with no signs of intradural progression (**Table 1**). Despite repeated extradural surgical revisions and prolonged treatment by several antibiotics, the infection relapsed recurrently after several months. The last revision was performed in September 2019 and the patient has been without infection relapse up to now. Moreover, the patient has been without any signs of tumor recurrence on MRI (**Figure 1E**) and in good clinical condition until December 2020 (disease-free survival of 61 months after the second resection of initial GBM recurrence), when the second GBM recurrence was detected by regular MRI (**Figure 1F**) and its further progression was confirmed by subsequent MRI scan 3 months later. Despite the radiotherapy treatment, the recurrence further progressed based on the latest MRI. Additionally, the patient is a passionate smoker and has been smoking approximately 20 cigarettes a day throughout the treatment.

**Table 1:** The details surrounding the patient’s treatment: HGG, high-grade glioma; STR, subtotal resection; GTR, gross-total resection.

	*Tumor localization*	*Number and extent of surgical treatments for HGG (time relapse)*	*Histological examination*	*Infectious complication/number of surgical treatments for infectious complications (etiology/antibiotic resistance)*	*ATB treatment (duration)*	*Outcome*
***Case 1***	Left parietal lobe	1. HGG resection (STR) 2. HGG resection after 5 months (GTR)	Glioblastoma WHO G IV, IDH-wildtype, MGMT promoter hypermethylation	Osteomyelitis/4 Staphylococcus aureus/resistance to clindamycin and erythromycin	Biseptol (680 days) Cefuroxime (60 days) Linezolid (21 days) Ospamo (23 days)	disease-free survival of 61 months
***Case 2***	Left frontal lobe	1. HGG resection (GTR) 2. HGG resection after 6 years (GTR)	Anaplastic astrocytoma, WHO GIII, IDH-mutant	Osteomyelitis/2 Staphylococcus aureus/not resistant Pseudomonas aeruginosa/resistant to piperacillin+tazobactam and ciprofloxacin Klebsiella pneumoniae/resistant to chloramphenicol and Biseptol)	Biseptol (60 days) Ceftazidime (14 days)	disease-free survival of 152 months

## 3 Case report 2

**Figure 2:**
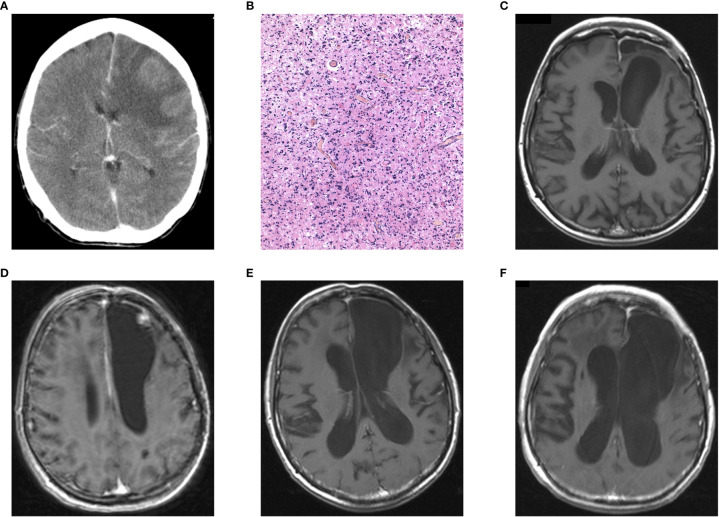
Case report 2: **(A)** preoperative postcontrast CT showing an intra-axial contrast-enhancing lesion in the left frontal lobe with perilesional edema, **(B)** histopathological finding of diffusely infiltrating astrocytoma with significant mitotic activity and cellular atypia classified as AA, IDH-mutant, WHO grade III (H&E, original magnification 100x), **(C)** postoperative MRI showing radical tumor resection, **(D)** AA recurrence 6 years after primary tumor resection, **(E)** 3 years follow-up MRI and **(F)** the last MRI with no signs of tumor recurrence.

A 53-year-old female patient with a history of epileptic seizures and the finding of a contrast-enhancing lesion in the left frontal lobe with perilesional edema on CT (**Figure 2A**) underwent radical tumor resection in 2003 with the histopathological result of AA, WHO grade III (**Figures 2B, C**). Subsequently, the patient without neurological symptoms underwent radiotherapy and adjuvant chemotherapy, which was discontinued after the second cycle due to intolerance (cachexia, anemia, thrombocytopenia). Despite incomplete oncological treatment, she was without any signs of tumor relapse on CT and subsequently MRI (available from 2004) for 6 years when MRI revealed a small enhancing lesion suspected to be a tumor recurrence (**Figure 2D**). A second surgery was performed and classified as a gross total resection (**Figure 2E**) with a histopathological diagnosis of AA IDH-mutant, WHO grade III. Two years after the second surgery, an infectious complication at the site of the surgical approach appeared. During the following 2 years, the patient underwent two surgical revisions with prolonged antibiotic treatment for osteomyelitis caused by Staphylococcus aureus, Pseudomonas aeruginosa, and Klebsiella pneumoniae (**Table 1**). Despite no tumor relapse on MRI (**Figure 2F**), the patient died of a respiratory infection 14 years (152 months) after the initial diagnosis of AA.

## 4 Discussion

Despite the general progression in the treatment of oncology diseases, the prognosis of patients suffering from HGG remains dismal with a median OS of 14-16 months and 3-5 years in GBM, IDH-wildtype, WHO grade IV and AA, IDH-mutant, WHO grade III, respectively (1, 2). Chronic wound infection is usually considered a severe complication in patients after HGG resection. Evidence from the literature to date is sparse and does not provide a clear conclusion on whether or not the postoperative infection affects survival in patients with HGG (7, 8). However, several case reports have been published of patients with a local wound infection after HGG resection and long-term survival (7, 9–11). Below, we discuss two cases of patients with long-term survival after treatment for GBM, IDH-wildtype, WHO grade IV (disease-free survival of 61 months after the second resection of first GBM recurrence), and AA, IDH-mutant, WHO grade III (OS 152 months) both cases were reclassified based on the WHO classification 2016 (2). In both cases, the chronic bacterial wound infection occurred after the second surgery and manifested as chronic osteomyelitis.

### 4.1 Impact of local wound infection on outcome in GBM, experimental and clinical findings

The experimental studies using intracranially implanted GBM cells and intra-tumoral bacterial lipopolysaccharide (LPS), a potent PAMP administration, proved increased survival time and tumor regression in animal models (12–14).

Despite promising experimental studies, comparing animal experiments with clinical studies remains problematic. Several factors may play a role in the severity of surgical wound infection. These factors include the time course and site of infection or the type of microorganism responsible for the infection. Therefore, there is considerable variation in conclusions between some studies. Regarding the early surgical wound complications, a study comprising 3748 patients after GBM resection did not show any profit from the bacterial site infection that occurred till a month after surgery. Such a complication was not associated with better outcomes and longer PFS (8).

Similarly, unplanned readmissions within 30 days after primary GBM resection for postoperative complications, including infectious, neurological, and thromboembolic, were associated with worse outcomes. In these patients, the median OS was shorter by 9 months compared to patients without postoperative infectious complications (15). A multicenter retrospective study from Salle et al. supported the assumption that surgical site infection after initial GBM resection leads to shorter OS. In these cases, the mean time between surgery for infection was 55 days (16). Moreover, Bohman et al. found in a single-center study with 382 patients that postoperative infection did not confer any survival advantage in GBM patients. However, subgroup analyses showed a non-significant advantage in mean survival in patients whose infection occurred late after HGG resection, as well as in patients with deep S. aureus infections (7). Based on these studies mentioned above, it can be assumed that the time between surgery and the onset of infection may play a role in OS in patients with HGG. However, it seems that etiologic agents may also play a particular role in the impact of infection on OS. In 2010, Bonis et al. had a significantly longer median survival time (30 months) in patients with bacterial infection when compared with patients without infection (15 months) with a high prevalence of S. aureus (60%). This was a single-center study with 197 patients with about 5 percent of infectious complications after primary surgery for malignant brain tumors, including grade 3 astrocytomas and GBMs (10).

In addition to the studies mentioned above, individual cases presenting the impact of bacterial infection on outcomes in patients after HGG resection have been described. A case report describing the infection of the Ommaya reservoir with S. aureus 3 years after primary GBM resection showed no recurrence of the tumor for 6 years after the onset of the infection (17). Bowles et al. referred to long-term survival in 4 cases after glioma resection followed by bacterial infection. In these cases, the authors suggested that tumor suppression may be potentiated by the immune response as well as the direct oncolytic effect of bacteria (18). This suggestion is supported by the finding of non-recurrence of GBM 4 years after tumor resection and treatment in a patient with subdural fluid collection positive for S. epidermidis (19).

### 4.2 Immune suppression in GBM

Another proposed mechanism is the stimulation patient’s immune response within or near the tumor bed while avoiding the systemic response (6, 11). It is well known that GBM induces tumor-associated immune suppression both within its microenvironment as well as systematically (20). Glioma cells, along with microglia, are potent to produce immunosuppressive factors to inhibit T-cell proliferation and stimulate T-regs (21, 22). At the same time, GBM induces systemic immunosuppression by reducing T-cell activity with preserved B-cell activity (23). GBM cancer stem cells are characterized by weak expression of MHC, and co-stimulatory molecules contribute to defective immunogenicity (14). Moreover, the expression of anti-inflammatory molecules like IL-10, PDL1, and FAS ligands by glioma-infiltrating cells probably reduces tumor immunogenicity (24).

### 4.3 The influence of microorganisms on the microenvironment in GBM

A growing body of literature has been describing both experimental as well as clinical evidence for complete or partial reduction of tumor cell growth by microorganisms in their close vicinity. During the microbial infection, the host cells recognize so-called pathogen-associated molecular patterns (PAMPs) present in microorganisms and alert the innate immune system (25). In animal models, inactivated bacteria experimentally inoculated into the tumor triggered an influx of macrophages, CD4+, and CD8+ T-cells, thus, stimulating effective anti-tumor response (26, 27). Another study described the effect of directly invading bacteria *via* activating the stimulator of IFN genes (STING), contributing to anti-tumor immunity by re-educating tumor-supportive M2 macrophages towards proinflammatory M1 phenotype and stimulating T-cell influx and action (28, 29). The phenotypic changes from M2 to M1 proinflammatory macrophages are supported by the activation of Toll-like receptor 9 (TLR9), which recognizes microbial products and initiates a complex immune response leading to the elimination of invading microorganisms. These changes include the secretion of proinflammatory molecules such as IL‐1, TNF‐α, ROS, and NO, which are able to affect tumor cells’ proliferation, migration, and invasiveness (29). This antitumoral effect was probably caused by the activation of toll-like receptor 4 (TLR4) as well as TLR9, which resulted in the activation of microglia and inflammatory cells at the site of the tumor process (13, 29). Moreover, depending on TLR4 signaling, *in vitro* LPS stimulation of GBM cells for 6 hours resulted in increased expression of proinflammatory molecules like MHC-I, MHC-II, CD80, CD86, CXCL10, TNF-α, IL-6, and down-regulation of the anti-inflammatory cytokine IL-10 (30).

Interestingly, *in vitro* study using GBM cells treated with S. aureus enterotoxin B (SEB) suggests another anti-tumor mechanism of bacterial infection. It was found that SEB can decrease smad2/3 expression in GBM cells leading to down-regulated TGF-β signaling and the reduction of tumor cell proliferation (31). Interestingly, there is some evidence that SEB has the ability to induce FasL/Fas (CD95L/CD95) mediated cytolysis through the CD8+ cytolytic T lymphocytes (32).

### 4.4 Other factors affecting GBM microenvironment

Apart from the possible impact of the bacterial infection on survival in both presented cases of HGG patients, some other related factors should be mentioned, such as antibiotic treatment, smoking, and the metabolic demands of bacteria.

There is some evidence that antibiotics targeting mitochondria can effectively eradicate cancer stem cells across multiple tumor types, including GBM (33). In this regard, the antibiotics with anti-tumor activity represent the erythromycins, tetracyclines, glycylcyclines, chloramphenicol, and an anti-parasitic drug-like pyrvinium pamoate. However, none of these antibiotics have been used in treating our patients.

Another considered etiopathogenic factor was nicotine consumption in our patient suffering from GBM because she was a heavy smoker. However, the recent review dealing with nicotine-containing products in patients treated with GBM suggested that nicotine has the potential not only to promote tumor growth but also to reduce the effectiveness of chemotherapeutic agents used in the treatment of HGG (34). On the other hand, *in vitro* studies on human glioma and glioblastoma cell lines showed nicotine dose-dependently cytotoxicity, which was probably caused by a rapid increase in the intracellular calcium concentration (19). Tobacco consumption was also associated with the loss of MGMT gene expression (35). The MGMT gene encodes a DNA-repair protein that removes alkyl groups from the O6 position of guanine, an important site of DNA alkylation, and contributes to the drug-resistant phenotype of GBM cells (36).

Several theories have been proposed to explain how bacteria affect the microenvironment around the GBM cells. One of the possible mechanisms is the sequestration of nutrients needed for bacterial cells leading to a limitation of resources for the high metabolic demands of GBM cells (6).

Despite these encouraging case reports, postoperative wound infection is still considered a serious condition, and randomized trials cannot be performed. However, bacterial toxins in different variants are laboratory tested as a new strategy for the treatment of different brain pathologies, including brain tumors. Our findings, therefore, justify the need for experimental studies focused on the interaction of bacterial components or toxins, the immune system, and glioma cells.

## 5 Conclusions

Although infection is considered a severe postoperative complication after HGG resection, it is possible that its chronic indolent course in some patients can contribute to their unexpectedly prolonged OS. We cannot present a precise mechanism that could be responsible for prolonged OS in our two patients but delayed surgical infection could be a mutual element in probably multifactorial etiology.

## Data availability statement

The original contributions presented in the study are included in the article/Supplementary Material. Further inquiries can be directed to the corresponding author.

## Ethics statement

Ethical review and approval was not required for the study on human participants in accordance with the local legislation and institutional requirements. The patients/participants provided their written informed consent to participate in this study.

## Author contributions

Sample acquisition: ZM, RJ, and PS. Sample processing: MiH and MaH. Data acquisition: PS, MiH, PP, and RL. Data analysis: PS, MiH, MaH, PP, RL, and HV. Data interpretation: PS, MiH, MaH, ZM, and RJ. Study conception: PS, MiH, MaH, ZM, RJ, and HV. Manuscript drafting and revision: PS, MiH, MaH, ZM, RJ, and PP. All authors read and approved the final manuscript.
